# Investigation of antibiotic resistance against pathogens isolated from respiratory samples in intensive care units after SARS-CoV-2 pandemic

**DOI:** 10.1186/s12879-025-12140-6

**Published:** 2025-12-03

**Authors:** Ayşe Çapar, Derya Özyiğitoğlu, Şeyma Başlılar

**Affiliations:** 1 Anaesthesiology and Intensive Care Medicine, Sultan Abdulhamid Han Training and Research Hospital, Selimiye Mh., Tıbbiye Cd., Üsküdar/İstanbul, 34668 Turkey; 2 Infectious Diseases and Clinical Microbiology, Sultan Abdulhamid Han Training and Research Hospital, Istanbul, Turkey; 3 Chest Diseases, Sultan Abdulhamid Han Training and Research Hospital, Istanbul, Turkey

**Keywords:** Antimicrobial resistance, Healthcare-associated infections, COVID-19 pandemic, Prevalence, Turkey

## Abstract

**Background:**

The COVID-19 pandemic has introduced major disruptions in clinical practices, including infection management strategies and antibiotic prescribing habits. This study aimed to investigate changes in the distribution of respiratory pathogens and their antimicrobial resistance (AMR) patterns in intensive care units (ICUs) before and after the onset of COVID-19 pandemic.

**Methods:**

In this retrospective cross-sectional study, 1,662 respiratory tract samples (bronchoalveolar lavage, deep tracheal aspirate, and sputum) collected from ICU patients at a tertiary hospital in Turkey between January 1, 2016 and December 31, 2024 were analyzed. The study compared bacterial isolates and resistance profiles between the pre-pandemic (January 2016–Feb 2020) and post-pandemic (Mar 2020–December 2024) periods.

**Results:**

*Acinetobacter baumannii* and *Klebsiella pneumoniae* remained the most frequently isolated pathogens throughout the study. Post-pandemic, resistance rates increased significantly for several antibiotics, particularly against *Acinetobacter baumannii* (gentamicin: 70.9% to 91.3%, *p* < 0.001; colistin: 3.2% to 12.3%, *p* < 0.001) and *Klebsiella pneumoniae* (carbapenems: *p* < 0.001; colistin: 23% to 62.7%, *p* < 0.001). For *Pseudomonas aeruginosa*, meropenem, piperacillin-tazobactam, and ceftazidime resistance rates increased significantly after the post-pandemic period (33.9% to 52.0%, *p* = 0.003; 50.4% to 65.1%, *p* = 0.011; 27.9% to 56.4%, *p* < 0.001, respectively). The changes in resistance rates of *Escherichia coli* were not statistically significant and also showed a reduced isolation rate post-pandemic (11.4% to 7.0%, *p* < 0.05). Among Gram-positive isolates, only *Enterococcus faecium* showed a significant increase in vancomycin resistance (50.0% vs. 0%, *p* = 0.046). In contrast, no significant findings were observed for methicillin resistance in *Staphylococcus aureus* (MRSA) and vancomycin resistance in *Enterococcus faecalis* (31.3% vs. 28.0%, *p* = 0.823; and 25% vs. 0%, *p* = 0.285, respectively). Mortality also increased significantly during the post-pandemic period (70.5% to 74.9%, *p* = 0.048), alongside a higher prevalence of comorbidities such as hypertension and coronary artery disease.

**Conclusions:**

The findings highlight a marked escalation in AMR following the COVID-19 pandemic, likely driven by increased empirical antibiotic use, prolonged ICU stays, expanded use of invasive procedures, and broader steroid administration. This study underscores the deepening AMR crisis in ICUs and provides critical data from Turkey that may support global antimicrobial stewardship efforts.

## Introduction

Antimicrobial resistance (AMR) is one of the most pressing health challenges in modern medicine. Combating infections caused by resistant microbes is especially difficult, often leading to increased treatment failures, longer hospital stays, and a significant economic strain on healthcare systems and governments [1]. For all these reasons, the World Health Organization (WHO) has recognized AMR as one of the top ten global health threats since 2015 and has declared it a public health emergency [2, 3]. Furthermore, a large-scale study published in 2021 reported that the number of confirmed deaths attributable to AMR was 1.14 million, while the number of deaths considered to be linked to AMR was 4.71 million [4].

The COVID-19 pandemic, which began in 2020, has led to notable changes in clinical protocols for infectious diseases worldwide. Challenges in distinguishing between viral and bacterial infections, the lack of effective antiviral treatments, widespread fear and uncertainty surrounding the disease, and the increased rates of Intensive Care Unit (ICU) admissions – where more resistant infections are commonly encountered than general wards - have collectively contributed to a substantial rise in empirical and broad spectrum antibiotic use, as antimicrobial therapy was perceived as a safer option [5–9]. Although numerous studies have suggested that frequent and unnecessary antibiotic use—particularly against Gram-negative microorganisms—may contribute to the development of resistance [6], definitive conclusions have yet to be established.

This study aims to evaluate the impact of the COVID-19 pandemic on AMR by comparing the resistance patterns of major pathogens isolated from respiratory samples before and after the pandemic in the ICU of a tertiary hospital in Istanbul. The findings are expected to contribute to the strengthening of AMR surveillance in Turkey and serve as a guide for the development of more effective antimicrobial stewardship strategies.

## Materials ve methods

This study was approved by the Scientific Research Ethics Committee of the Istanbul Health Sciences University Umraniye Training and Research Hospital (Approval Date: 11 July 2024; Approval No: B.10.1.TKH.4.34.H.GP.0.01/203). The study was conducted by the principles of the Declaration of Helsinki. As this was a retrospective study, the requirement for informed consent was waived.

In this retrospective cross-sectional study, we analyzed data from patients admitted to the Pulmonology ICU at Sultan Abdulhamid Han Training and Research Hospital between January 1, 2016, and December 31, 2024. The study compares two distinct periods: the pre-pandemic period (January 2016 – February 2020) and the post-pandemic period (March 2020 – December 2024).

### Patient and sample selection

A total of 1,662 respiratory tract samples from 1,646 patients were included in the study. The study population consisted of ICU patients with hospital-acquired pneumonia, aspiration pneumonia, chronic obstructive pulmonary disease (COPD) exacerbations, COVID-19 pneumonia, and sepsis. The included samples consisted of bronchoalveolar lavage (BAL), deep tracheal aspirate (DTA), and sputum specimens. Repeated isolations and samples lacking sufficient clinical data were excluded. When the same pathogen was isolated multiple times from respiratory specimens of the same patient, only the first isolation was used for AMR analysis. Demographic and clinical data of the included patients, along with comorbidities such as diabetes mellitus (DM), hypertension (HT), coronary artery disease (CAD), COPD, arrhythmias, congestive heart failure (CHF), and malignancy, were obtained from the hospital information management system. Additionally, patient survival following AMR infections was also recorded.

### Microbiological processing

Bacteria isolated from respiratory samples were identified using both conventional culture methods and automated systems, such as the VITEK 2 Compact. After proper transport to the laboratory, all samples underwent Gram staining to assess cellular content and microbial morphology, and sputum specimens were evaluated for quality. The samples were inoculated onto sheep blood agar, chocolate agar (incubated in 5% CO₂), and MacConkey agar. All plates were incubated at 35–37 °C under aerobic conditions, with chocolate agar evaluated in 5% CO₂. Cultures were examined after 18–24 h and extended up to 48 h if necessary. Antimicrobial susceptibility testing was performed in accordance with the Clinical and Laboratory Standards Institute (CLSI)-M100 guidelines and the European Committee on Antimicrobial Susceptibility Testing (EUCAST) recommendations.

The study examined the resistance patterns of the most commonly isolated respiratory pathogens, including four Gram-negative bacteria (*Acinetobacter baumannii*,* Klebsiella pneumoniae*,* Pseudomonas aeruginosa*, and *Escherichia coli*) and Gram-positive bacteria (*Staphylococcus aureus*,* Enterococcus faecium*, and *Enterococcus faecalis*). Susceptibility rates to a comprehensive panel of antimicrobial agents were determined by disk diffusion method, including commonly used antibiotics such as amikacin (30 µg), gentamicin (10 µg), tobramycin (10 µg), amoxicillin-clavulanic acid (20/10 µg), ampicillin (2 µg), ampicillin-sulbactam (10/10 µg), piperacillin-tazobactam (100/10 µg), cefazolin (30 µg), cefoxitin (30 µg), cefepime (30 µg), ceftazidime (30 µg), ceftriaxone (30 µg), ciprofloxacin (5 µg), levofloxacin (5 µg), trimethoprim-sulfamethoxazole (1.25/23.75 µg), ertapenem (10 µg), meropenem (10 µg), imipenem (10 µg), colistin (4 µg/ml), teicoplanin (30 µg), vancomycin (16 µg/ml), tigecycline (15 µg), benzylpenicillin (10 U), oxacillin (4 µg/ml), clindamycin (2 µg), and erythromycin (15 µg). All values (µg) refer to disk contents used in the disk diffusion method. However, for *Staphylococcus aureus*, resistance to oxacillin and vancomycin is determined by minimum inhibitory concentration (MIC) values (µg/mL). Vancomycin resistance in *Enterococci* is also assessed by the disk diffusion method, with a vancomycin disk containing 30 µg. Susceptibility to colistin was determined by the broth microdilution method (µg/mL).

### Statistical analysis

Data were analyzed using SPSS (Statistical Package for the Social Sciences) version 30. Continuous variables were presented as mean ± standard deviation, while categorical variables were expressed as frequencies and percentages (%). To assess differences between the pre-pandemic and post-pandemic periods, the Chi-square test or Fisher’s exact test was applied. For comparisons of continuous variables, the independent samples t-test or Mann-Whitney U test was used. A p-value of *< 0.05* was considered statistically significant.

## Results

### Descriptive statistics

Within the study, 1,662 ICU respiratory samples (bronchoalveolar lavage, deep tracheal aspirate, sputum) collected between January 1, 2016 and December 31, 2024 were retrospectively analyzed, including 683 pre-pandemic, 979 post-pandemic, and 150 (9.1%) from COVID-19 PCR-positive patients (Table 1).

**Table 1:** Comparison of clinical, demographic, and microbiological characteristics of patients before and after the COVID-19 pandemic

	Overall mean±sd or *n* (%)	Before COVID-19 Pandemic median (min-max) or *n* (%)	After COVID-19 Pandemic median (min-max) or *n* (%)	*p*-value
Age	71.63±15.45	74 (20–100)	75 (17–104)	*0.218*
**Gender** Male Female	1018 (61.8) 628 (38.2)	424 (62.6) 253 (37.4)	594 (61.3) 375 (38.7)	*0.585*
Exitus	1203 (73.1)	470 (70.5)	733 (74.9)	***0.048***
DM	479 (29.1)	184 (27.6)	295 (30,1)	*0.264*
HT	731 (44.4)	225 (33.7)	502 (51.7)	***< 0.001***
CAD	262 (15.9)	71 (10.6)	191 (19.5)	***< 0.001***
COPD	381 (23.1)	143 (21.4)	238 (24.3)	*0.175*
Arryhthmias	142 (8.6)	34 (5.1)	108 (11)	***< 0.001***
CHF	264 (16)	97 (14.5)	167 (17.1)	*0.172*
Malignancy	411 (25)	143 (21.4)	268 (27.4)	***0.006***
COVID (+) patients			150 (9.1)	
Number of the respiratory samples	1662 (100)	683 (41.1)	979 (58.9)	
**Pathogens** *Acinetobacter Baumanni* *Klebsiella Pneumoniae* *Pseudomonas Aeruginosa* *Escherichia Coli* *Staphilococcus Aureus* *Enterococcus Feacium* *Enterococcus Faecalis* *Stenotrophomonas Maltophilia* *Serratia Marcescens* *Proteus Mirabilis* *Candida Albicans* *Candida Parapisilosis* *Candida Tropicalis*	559 (33.6) 438 (26.4) 312 (18.8) 147 (8.8) 70 (4.2) 18 (1.1) 9 (0.5) 50 (3) 26 (1.6) 19 (1.1) 7 (0.4) 1 (0.1) 6 (0.4)	226 (33.1) 152 (22.3) 129 (18.9) 78 (11.4) 40 (5.9) 6 (0.9) 4 (0.6) 14 (2) 13 (1.9) 11 (1.6) 5 (0.7) 0 (0) 5 (0.7)	333 (34) 286 (29.2) 183 (18.7) 69 (7) 30 (3.1) 12 (1.2) 5 (0.5) 36 (3.7) 13 (1.3) 8 (0.8) 2 (0.2) 1 (0.1) 1 (0.1)	***< 0.001***

DM: Diabetes Mellitus, HT: Hypertension, CAD: Coronary Artery Diseases, COPD: Chronic Obstructive Pulmonary Disease, CHF: Congestive Heart Failure. Continuous variables were analyzed using the Mann–Whitney U test, while categorical variables were evaluated using the Chi-square test or Fisher’s exact test, as appropriate

Among 1,646 patients, 1,018 (61.8%) were male and 628 (38.2%) were female, with no significant difference in gender distribution between the pre- and post-pandemic periods (male: 62.6% vs. 61.3%, female: 37.4% vs. 38.7%, *p* = 0.585). The median age was 74 (20–100) before the pandemic and 75 (17–104) after; there is no significant difference in age (*p* = 0.218). In the post-pandemic period, HT (33.7% vs. 51.7%, *p* < 0.001), CAD (10.6% vs. 19.5%, *p* < 0.001), arrhythmia rate (5.1% vs. 11%, *p* < 0.001), and malignancy (21.4% vs. 27.4%, *p* = 0.006) were significantly more prevalent. No differences were observed in CHF, COPD, or DM (*p* > 0.05). Furthermore, when comparing mortality rates before and after the pandemic, a significantly larger proportion of patients died during the post-pandemic period (70.5% vs. 74.9%, *p* = 0.048) (Table 1). When COVID–19–positive patients were excluded and mortality rates were reexamined, no statistically significant difference was found between the pre-COVID-19 and post-COVID-19 periods (70.5% vs. 74.5%, *p* = 0.078).

### Gram-negative bacteria

#### Acinetobacter baumannii

Throughout the study period, *Acinetobacter baumannii* was the most frequently isolated pathogen, with an isolation rate of 33.1% in the pre-pandemic period and 34.0% in the post-pandemic period (*p* = 0.695).

**Fig. 1:**
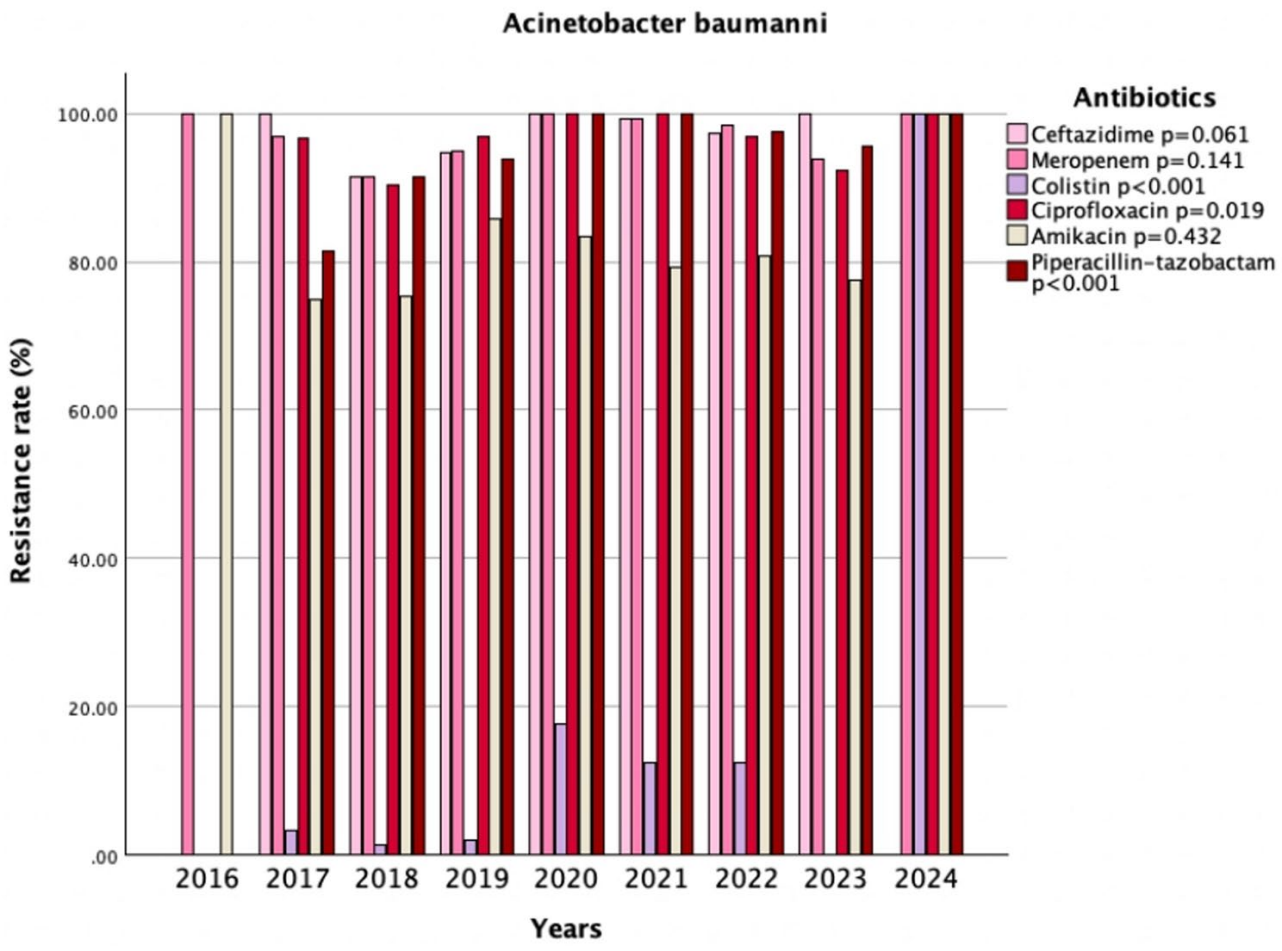
Trends in antimicrobial resistance patterns of *Acinetobacter baumannii* isolates (2016–2024)

In the post-pandemic period, gentamicin resistance increased significantly from 70.9% to 91.3% (*p* < 0.001). Similarly, significant increases were observed in resistance to colistin (3.2% vs. 12.3%, *p* < 0.001), piperacillin-tazobactam (91.7% vs. 98.2%, *p* < 0.001), ceftazidime (94.7% vs. 98.4%, *p* = 0.047), trimethoprim/sulfamethoxazole (85.4% vs. 93.9%, *p* = 0.001), and tigecycline (4.8% vs. 14.2%, *p* < 0.001). Although resistance to agents such as meropenem, imipenem, ciprofloxacin, and levofloxacin (94.6% vs. 97.8%, *p* = 0.078; 91.3% vs. 96.1%, *p* = 0.136; 95% vs. 97.3%, *p* = 0.234; 91.2% vs. 97.7%, *p* = 0.079 respectively) was already high in the pre-pandemic period and showed further increases after the pandemic, these changes did not reach statistical significance (Table 2). A statistically significant change over the years was observed in the AMR of *Acinetobacter baumannii* to colistin, ciprofloxacin, and piperacillin–tazobactam (*p* < 0.001, *p* = 0.019, *p* < 0.001, respectively) (Fig. 1).

**Table 2:** Antibiotic resistance evaluation of *Acinetobacter baumannii* before and after the COVID-19 pandemic

	Before COVID-19 Pandemic (*n* %) *n* = 226	After COVID-19 Pandemic (*n* %) *n* = 333	*p*-value
Amikacin (30 µg)	183 (81)	267 (80.2)	*0.816*
Gentamicin (10 µg)	158 (70.9)	230 (91.3)	***< 0.001***
Meropenem (10 µg)	211 (94.6)	314 (97.8)	*0.078*
Imipenem (10 µg)	73 (91.3)	174 (96.1)	*0.136*
Colistin (4 µg/mL)	7 (3.2)	25 (12.3)	***< 0.001***
Piperacillin-tazobactam (100/10 µg)	200 (91.7)	322 (98.2)	***< 0.001***
Cefepime (30 µg)	7 (77.8)	4 (10.3)	***< 0.001***
Ceftazidime (30 µg)	198 (94.7)	252 (98.4)	***0.047***
Ceftriaxone (30 µg)	4 (100)	2 (40)	*0.167*
Ciprofloxacin (5 µg)	207 (95)	322 (97.3)	*0.234*
Levofloxacin (5 µg)	62 (91.2)	86 (97.7)	*0.079*
Trimethoprim/ Sulfamethoxazole (1.25/23.75 µg)	187 (85.4)	310 (93.9)	***0.001***
Tigecycline (15 µg)	10 (4.8)	46 (14.2)	***< 0.001***

All values (µg) refer to disk contents used in the disk diffusion method; Colistin susceptibility was evaluated by broth microdilution method (µg/mL); Antibiotic resistance rates before and after the COVID-19 pandemic were compared using the Chi-square test or Fisher’s exact test, as appropriate

#### Klebsialla pneomoniae

**Fig. 2:**
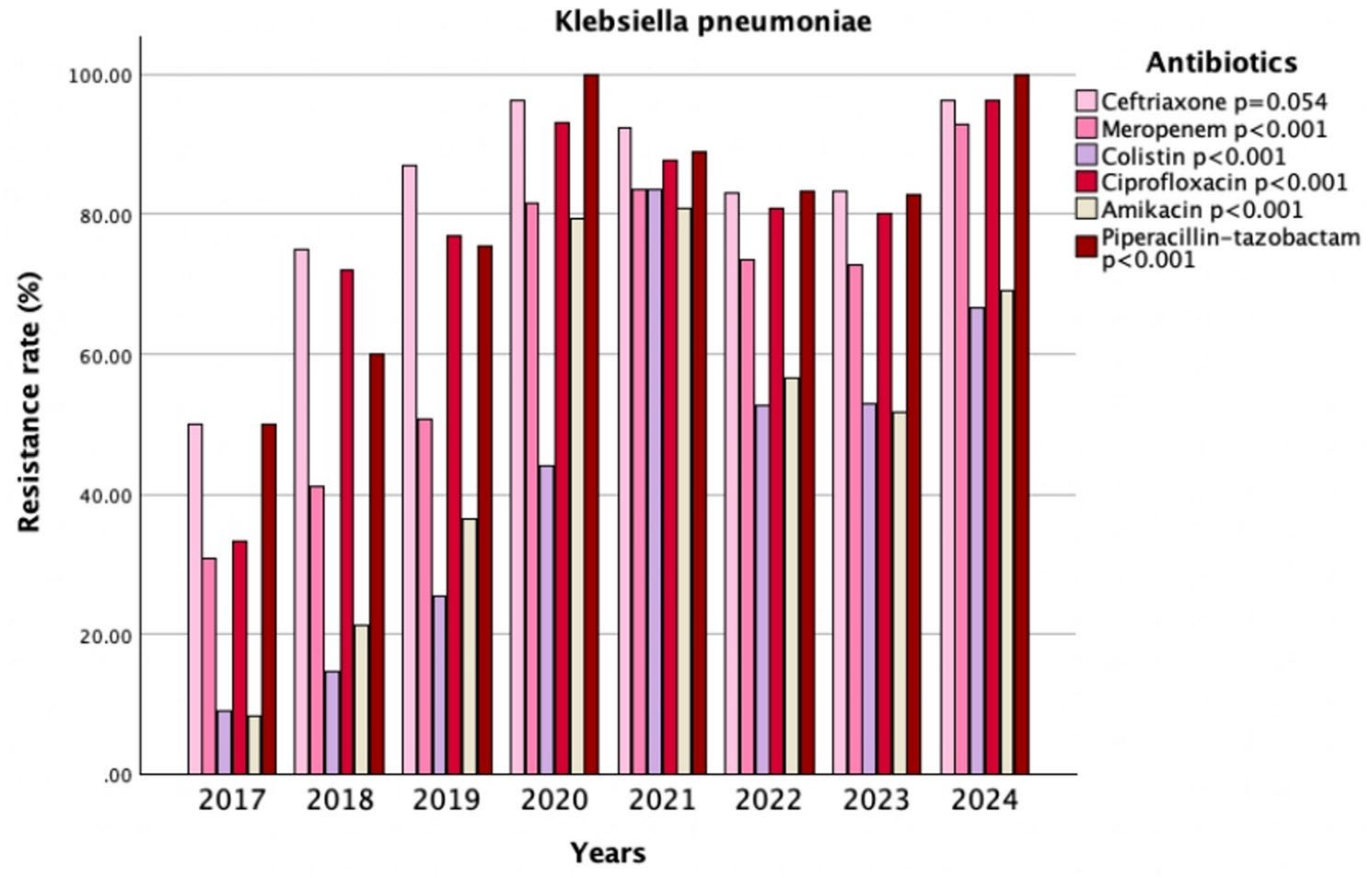
Trends in antimicrobial resistance patterns of *Klebsiella pneumoniae* isolates (2017–2024)

The isolation rate of *Klebsiella pneumoniae* rose from 22.3% to 29.2% after the pandemic (*p* = 0.002). According to the AMR analysis, *Klebsiella pneumoniae* isolates in the post-pandemic period showed significant increases in resistance to amikacin (63.5% vs. 35.6%, *p* < 0.001), gentamicin (71.1% vs. 54.7%, *p* = 0.002), carbapenems (ertapenem, 80.4% vs. 63.6% *p* < 0.001; meropenem, 77.8% vs. 50.3% *p* < 0.001; imipenem, 71.7% vs. 32.6% *p* < 0.001), and colistin (62.7% vs. 23%, *p* < 0.001). A marked rise in resistance rates was also observed for broad-spectrum antibiotics, including ceftazidime (89.1% vs. 81.3%, *p* = 0.037), cefepime (90.4% vs. 80.7%, *p* = 0.009), piperacillin-tazobactam (86.7% vs. 70%, *p* < 0.001), trimethoprim-sulfamethoxazole (77.3% vs. 61.1%, *p* < 0.001), and tigecycline (85.1% vs. 27.9%, *p* < 0.001). Similarly, resistance to quinolones—levofloxacin and ciprofloxacin—was significantly higher in the post-pandemic period (100% vs. 58.3%, *p* < 0.001; 85.1% vs. 72.7%, *p* = 0.002, respectively) (Table 3). When evaluating AMR in *Klebsiella pneumoniae* over the years, significant changes were observed in resistance to meropenem, colistin, ciprofloxacin, amikacin, and piperacillin–tazobactam (*p* < 0.001, for both) (Fig. 2).

**Table 3:** Antibiotic resistance evaluation of *Klebsiella pneumoniae* before and after the COVID-19 pandemic

	Before COVID-19 Pandemic (*n* %) *n* = 152	After COVID-19 Pandemic (*n* %) *n* = 286	*p*-value
Amoxicillin/clavulanic acid (20/10 µg)	102 (85.7)	246 (88.8)	*0.486*
Ampicillin (2 µg)	118 (100)	277 (99.6)	*1.0*
Amikacin (30 µg)	53 (35.6)	176 (63.5)	***< 0.001***
Gentamicin (10 µg)	81 (54.7)	140 (71.1)	***0.002***
Ertapenem (10 µg)	70 (63.6)	217 (80.4)	***< 0.001***
Meropenem (10 µg)	73 (50.3)	200 (77.8)	***< 0.001***
Imipenem (10 µg)	14 (32.6)	71 (71.7)	***< 0.001***
Colistin (4 µg/mL)	31 (23)	101 (62.7)	***< 0.001***
Ciprofloxacin (5 µg)	109 (72.7)	240 (85.1)	***0.002***
Levofloxacin (5 µg)	14 (58.3)	21 (100)	***< 0.001***
Piperacillin-tazobactam (100/10µg)	98 (70)	242 (86.7)	***< 0.001***
Cefazolin (30 µg)	98 (94.2)	183 (93.8)	*1.0*
Cefoxitin (30 µg)	71 (67)	218 (79.3)	***0.012***
Ceftazidime (30 µg)	117 (81.3)	246 (89.1)	***0.037***
Ceftriaxone (30 µg)	97 (83.6)	237 (87.5)	*0.39*
Cefepime (30 µg)	113 (80.7)	244 (90.4)	***0.009***
Trimethoprim/ Sulfamethoxazole (1.25/23.75 µg)	91 (61.1)	215 (77.3)	***< 0.001***
Tigecycline (15 µg)	38 (27.9)	63 (85.1)	***< 0.001***

All values (µg) refer to disk contents used in the disk diffusion method; Colistin susceptibility was evaluated by broth microdilution method (µg/mL); Antibiotic resistance rates before and after the COVID-19 pandemic were compared using the Chi-square test or Fisher’s exact test, as appropriate

#### Pseudomonas aeruginosa

The frequency of *Pseudomonas aeruginosa* isolates remained similar between the pre-pandemic and post-pandemic periods, at 18.9% and 18.7%, respectively (*p* = 0.920).

**Fig. 3:**
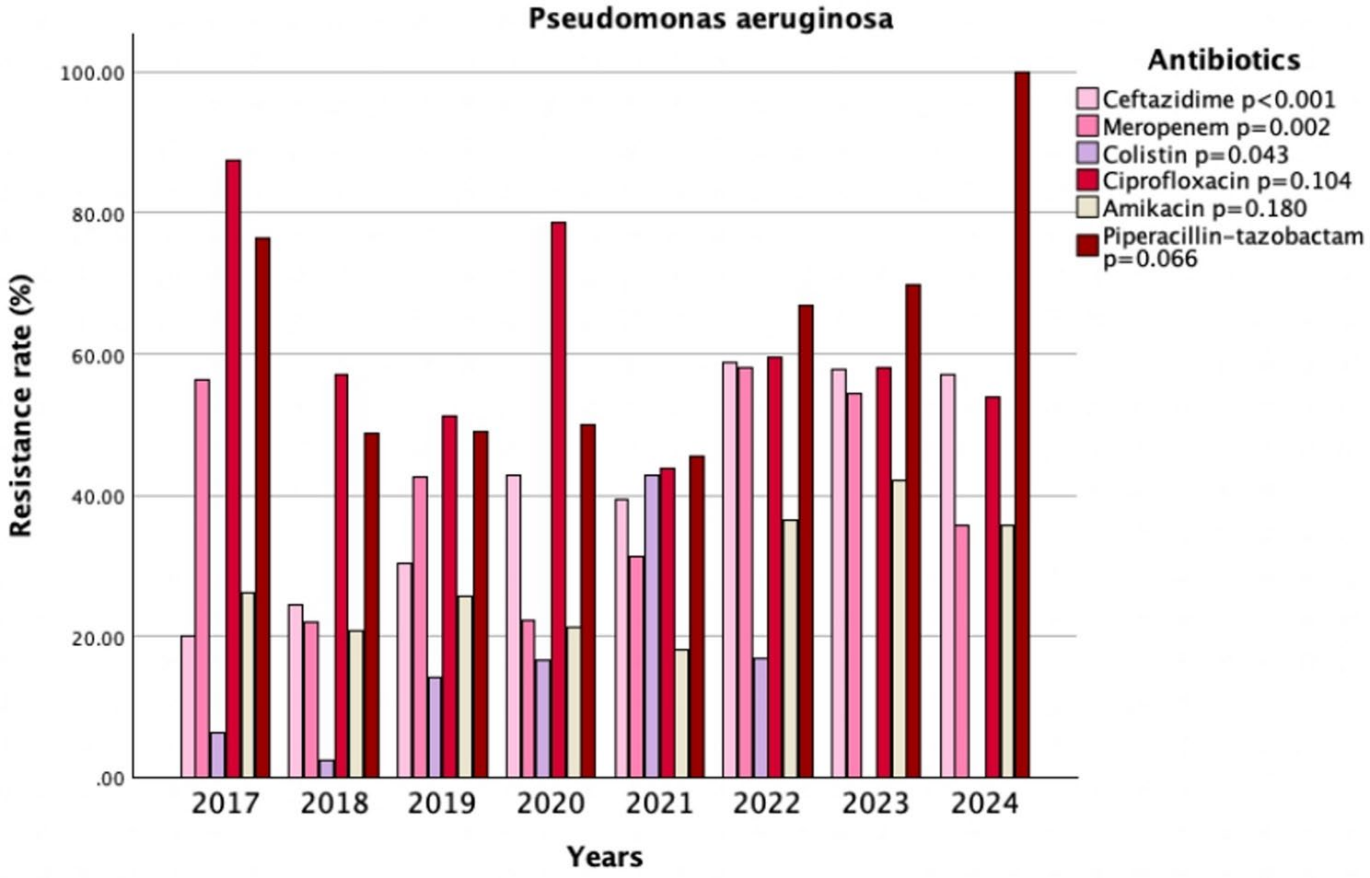
Trends in antimicrobial resistance patterns of *Pseudomonas aeruginosa* isolates (2017–2024)

Notably, resistance rates increased significantly for meropenem (52.0% vs. 33.9%, *p* = 0.003), ceftazidime (56.4% vs. 27.9%, *p* < 0.001), cefepime (57.2% vs. 40.5%, *p* = 0.005), piperacillin-tazobactam (65.1% vs. 50.4%, *p* = 0.011), and amikacin (35.7% vs. 22.4%, *p* = 0.013). Resistance to ciprofloxacin and levofloxacin remained similar between the pre-pandemic and post-pandemic periods (59% vs. 57.6%, *p* = 0.81; 62.2% vs. 57.4%, *p* = 0.76). All isolates were susceptible to tigecycline (Table 4). Over the years, when evaluating AMR in *Pseudomonas aeruginosa*, significant changes in resistance to ceftazidime, meropenem, and colistin were observed (*p* < 0.001, *p* = 0.002, *p* = 0.043, respectively) (Fig. 3).

**Table 4:** Antibiotic resistance evaluation of *Pseudomonas aeruginosa* before and after the COVID-19 pandemic

	Before COVID-19 Pandemic (*n* %) *n* = 129	After COVID-19 Pandemic (*n* %) *n* = 183	*p*-value
Amikacin (30 µg)	28 (22.4)	65 (35.7)	***0.013***
Gentamicin (10 µg)	32 (26.9)	3 (10.3)	*0.102*
Meropenem (10 µg)	38 (33.9)	92 (52)	***0.003***
Imipenem (10 µg)	26 (46.4)	86 (53.8)	*0.345*
Colistin (4 µg/mL)	8 (8.2)	11 (17.7)	*0.116*
Piperacillin-tazobactam (100/10 µg)	61 (50.4)	114 (65.1)	***0.011***
Ceftazidime (30 µg)	34 (27.9)	102 (56.4)	***< 0.001***
Cefepime (30 µg)	47 (40.5)	99 (57.2)	***0.005***
Ciprofloxacin (5 µg)	72 (59)	102 (57.6)	*0.81*
Levofloxacin (5 µg)	23 (62.2)	58 (57.4)	*0.76*
Tobramycin (10 µg)	11 (22.9)	33 (30.8)	*0.41*

All values (µg) refer to disk contents used in the disk diffusion method; Colistin susceptibility was evaluated by broth microdilution method (µ/mL); Antibiotic resistance rates before and after the COVID-19 pandemic were compared using the Chi-square test or Fisher’s exact test, as appropriate

#### Escherichia coli

The proportion of *Escherichia coli* isolates was lower in the post-pandemic period (7.0% vs. 11.4%, *p* = 0.02).

**Fig. 4:**
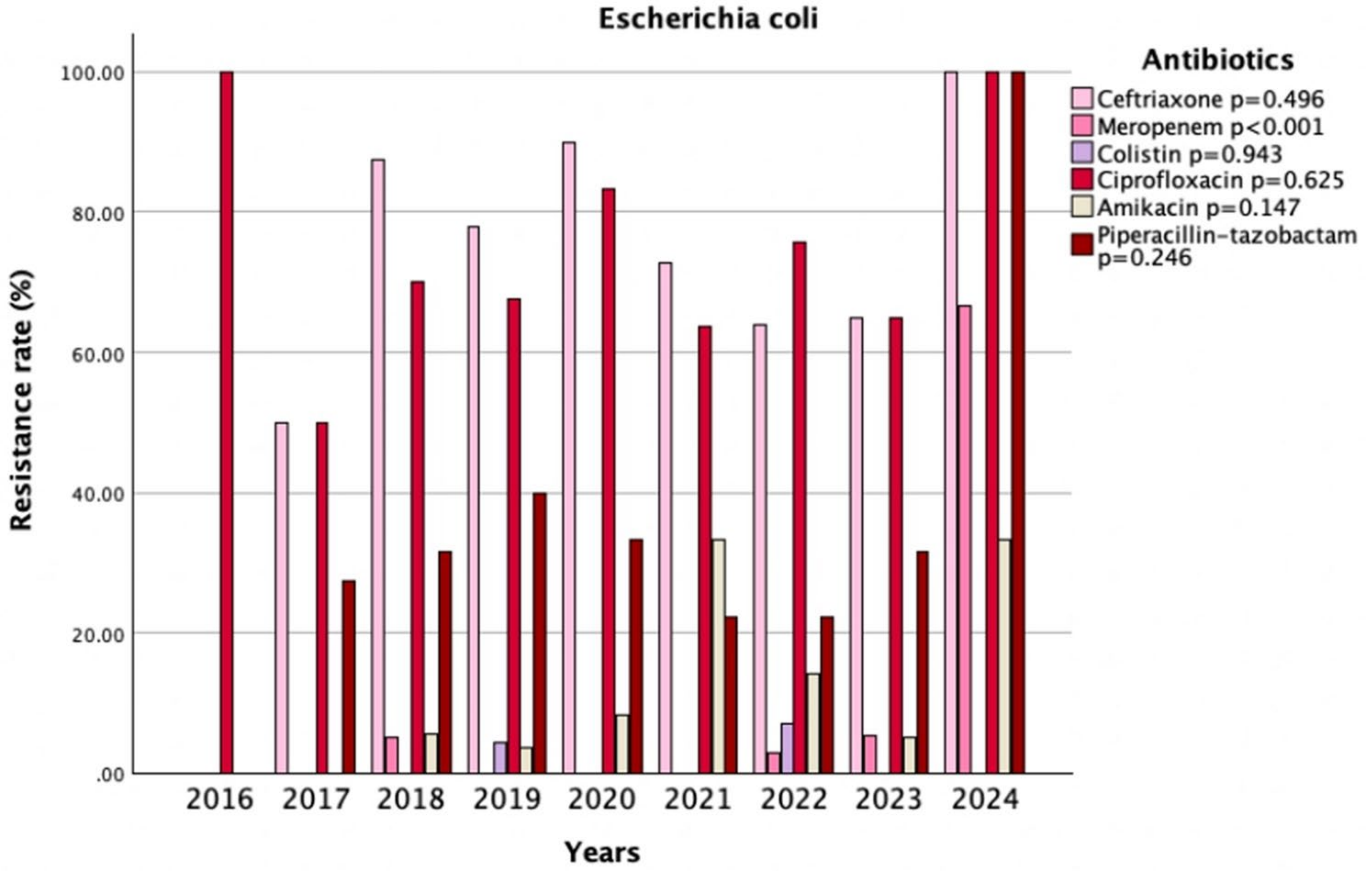
Trends in antimicrobial resistance patterns of *Escherichia coli* isolates (2016–2024)

In the AMR analysis, no significant overall changes were observed. Resistance rates to amikacin, meropenem, and ciprofloxacin stayed relatively stable before and after the pandemic (4.2% vs. 15.4%, *p* = 0.052; 1.4% vs. 2.3%, *p* = 0.183; 70.1% vs. 71%, *p* = 1.0). Although not statistically significant, a decreasing trend in resistance to cephalosporins was observed in the post-pandemic period (cefazolin: 88.9% vs. 80.4%, *p* = 0.408; cefoxitin: 31.1% vs. 19.4%, *p* = 0.232; ceftazidime: 72.2% vs. 66.2%, *p* = 0.559; ceftriaxone: 81.6% vs. 66.2%, *p* = 0.101; cefepime: 64.8% vs. 57.4%, *p* = 0.489) (Table 5). Among all the antibiotics tested against *Escherichia coli* over the years, only resistance to meropenem changed significantly over time (*p* < 0.001) (Fig. 4).

**Table 5:** Antibiotic resistance evaluation of *Escherichia coli* before and after the COVID-19 pandemic

	Before COVID-19 Pandemic (*n* %) *n* = 78	After COVID-19 Pandemic (*n* %) *n* = 69	*p*-value
Amoxicillin/clavulanic acid (20/10 µg)	40 (75.5)	42 (61.8)	*0.16*
Ampicillin (2 µg)	49 (92.5)	61 (89.7)	*0.754*
Amikacin (30 µg)	3 (4.2)	10 (15.4)	***0.052***
Gentamicin (10 µg)	26 (34.2)	13 (26.5)	*0.48*
Meropenem (10 µg)	1 (1.4)	4 (2.3)	*0.183*
Imipenem (10 µg)	1 (3.8)	2 (6.7)	*1.00*
Ertapenem (10 µg)	5 (10.4)	6 (10)	*1.00*
Colistin (4 µg/mL)	1 (1.8)	1 (6.3)	*0.397*
Piperacillin-tazobactam (100/10 µg)	21 (31.8)	19 (29.7)	*0.85*
Cefazolin (30 µg)	40 (88.9)	37 (80.4)	*0.408*
Cefoxitin (30 µg)	14 (31.1)	13 (19.4)	*0.232*
Ceftazidime (30 µg)	52 (72.2)	43 (66.2)	*0.559*
Ceftriaxone (30 µg)	40 (81.6)	45 (66.2)	*0.101*
Cefepime (30 µg)	46 (64.8)	35 (57.4)	*0.489*
Ciprofloxacin (5 µg)	54 (70.1)	49 (71)	*1.0*
Levofloxacin (5 µg)	14 (73.7)	4 (100)	*0.539*
Trimethoprim/ Sulfamethoxazole (1.25/23.75 µg)	45 (59.2)	46 (67.6)	*0.382*
Tigecycline (15 µg)	3 (4.7)	6 (10.5)	*0.304*

All values (µg) refer to disk contents used in the disk diffusion method; Colistin susceptibility was evaluated by broth microdilution method (µg/mL); Antibiotic resistance rates before and after the COVID-19 pandemic were compared using the Chi-square test or Fisher’s exact test, as appropriate

#### Gram-positive bacteria

**Fig. 5:**
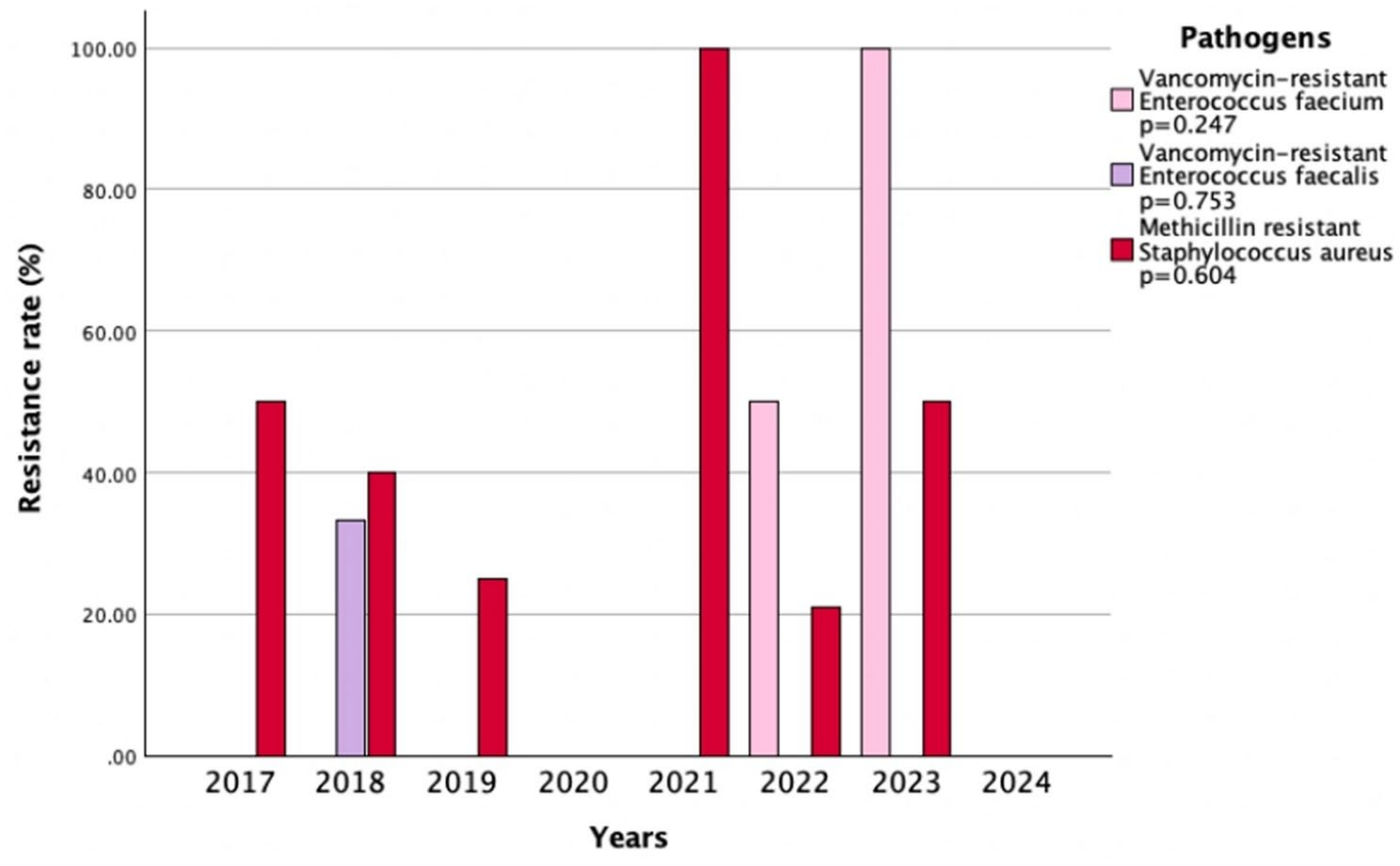
Trends in antimicrobial resistance rates of Gram-positive bacteria (2017–2024)

The Gram-positive bacteria were present in 5.8% of the samples. The isolation rate of *Staphylococcus aureus* was lower in the post-pandemic period (3.1% vs. 5.9%, *p* = 0.005). The methicillin resistance rate in these isolates remained similar before and after the pandemic (31.3% vs. 28%, *p* = 0.823). *Enterococcus* species were isolated infrequently and at similar rates before and after pandemics: 0.6% vs. 0.5%, *p* = 0.838 for *Enterococcus faecalis* and 0.9% vs. 1.2%, *p* = 0.501 for *Enterococcus faecium*. No significant rise in resistance to ciprofloxacin, trimethoprim-sulfamethoxazole, or vancomycin was observed among *Enterococcus faecalis* isolates. In *Enterococcus faecium*, resistance rates to teicoplanin (*p* = 0.006) and vancomycin (*p* = 0.046) increased significantly in the post-pandemic period (Table 6). When analyzing the changes over the years, no significant variations were found in vancomycin-resistant *Enterococcus faecium*, vancomycin-resistant *Enterococcus faecalis*, or methicillin-resistant *Staphylococcus aureus* strains (*p* = 0.247, *p* = 0.753, *p* = 0.604, respectively) (Fig. 5).

**Table 6:** Antibiotic resistance evaluation of *Staphylococcus aureus*,* Enterococcus faecalis* and *Enterococcus faecium* before and after the COVID-19 pandemic

	Staphylococcus Aureus			Enterococcus Faecalis			Enterococcus Faecium		
	Before COVID-19 Pandemic (*n* %) *n* = 40	After COVID-19 Pandemic (*n* %) *n* = 30	*p*-value	Before COVID-19 Pandemic (*n* %) *n* = 4	After COVID-19 Pandemic (*n* %) *n* = 5	*p*-value	Before COVID-19 Pandemic (*n* %) *n* = 6	After COVID-19 Pandemic (*n* %) *n* = 12	*p*-value
Ciprofloxacin (5 µg)	10 (27)	0 (0)	*0.146*	3 (75)	1 (50)	*0.54*	6 (100)	3 (42.9)	***0.026***
Trimethoprim/ Sulfamethoxazole (1.25/23.75 µg)	3 (7.5)	0 (0)	*0.125*	4 (100)	4 (80)	*0.343*	3 (50)	3 (33.3)	*0.519*
Teicoplanin (30 µg)	5 (16.7)	0 (0)	***0.054***	1 (25)	0 (0)	*0.35*	0 (0)	4 (80)	***0.006***
Vancomycin. (16 µg/mL)**	0 (0)	0 (0)	*NA*	1 (25)	0 (0)	*0.285*	0 (0)	3 (50)	***0.046***
Benzylpenicillin (10 U)	9 (100)	13 (86.7)	*0.253*	*	0 (0)	*NA*	*	0 (0)	*NA*
Oxacillin (4 µg/mL)**	5 (31.3)	7 (28)	*0.823*	*	*	*NA*	*	*	*NA*
Clindamycin (2 µg)	12 (31.6)	10 (33.3)	*0.878*	*	0 (0)	*NA*	2 (100)	1 (33.3)	*0.136*
Erythromycin (15 µg)	4 (50)	1 (7.1)	***0.021***	*	0 (0)	*NA*	*	0 (0)	*NA*

NA: Not Applicable; *Not performed; **For Staphylococcus aureus, vancomycin and oxacillin susceptibility was determined solely by minimum inhibitory concentration (MIC) testing, as disk diffusion is not recommended for this species according to CLSI guidelines (µg/mL); All values (µg) refer to the contents on the disk used in the disk diffusion method; this method can determine vancomycin resistance in enterococci, and the vancomycin content on the disk is 30 µg. Antibiotic resistance rates before and after the COVID-19 pandemic were compared using the Chi-square test or Fisher’s exact test, as appropriate

## Discussion

This retrospective study was conducted in the respiratory ICUs of a tertiary hospital in Turkey, comparing the distribution of major pathogens isolated from respiratory tract samples and changes in AMR patterns between January 1, 2016 and December 31, 2024, focusing on the impact of the COVID-19 pandemic. The results showed significant shifts in both the distribution of infectious agents and resistance rates after the pandemic. Notably, changes were observed in both Gram-negative and Gram-positive pathogens, with a marked increase in *Klebsiella pneumoniae* and *Acinetobacter baumannii* isolates following the pandemic. And these two bacteria also showed increasing AMR in 2020 and 2021 when evaluated over time.

In the post-pandemic period, there was a notable rise in comorbidities such as HT, CAD, arrhythmia, and malignancy. These conditions are believed to compromise the host’s immune defenses, increasing the likelihood of severe infections and the development of resistant pathogens. At the same time, mortality rates also increased significantly (from 70.5% to 74.9%; *p* = 0.048), possibly partly due to the rise in AMR. Supporting this link, even after excluding COVID-positive patients, there was a non-significant upward trend in AMR and mortality (70.5% vs. 74.5%, *p* = 0.078), suggesting that secondary infections during the pandemic may have contributed to the excess deaths.

COVID-19 can directly predispose patients to secondary bacterial infections by causing inflammatory dysregulation and cytokine storms [10]. During the pandemic, prolonged hospital and ICU stays, increased use of invasive procedures, and more frequent application of mechanical ventilation markedly elevated the risk of secondary bacterial infections [8]. This increase cannot be attributed solely to the pandemic, but may also reflect advances in intensive care that enabled the broader use of invasive procedures. At the same time, the rapid expansion of ICU capacity after the pandemic—with the involvement of less experienced staff and the lack of legally defined admission criteria in Turkey—probably compromised infection control, leading to secondary infections. Consistent with this, our study revealed a significant increase in diseases caused by *Klebsiella pneumoniae* and *Acinetobacter baumannii* during the post-pandemic period.

In COVID-19 treatment, glucocorticoids have been widely used as adjunct therapy, reducing excessive lung inflammation, limiting tissue damage, and lowering 28-day mortality, especially in severe cases [8]. However, some studies have reported administering high-dose glucocorticoids (e.g., 250 mg/day or 1 g/day) to patients with COVID-19 pneumonia [11, 12]. While steroids were previously used more cautiously and selectively in the management of pneumonia, their broader and more liberal use during the pandemic may have facilitated the development of secondary bacterial infections. This shift may also have indirectly contributed to changes in AMR patterns.

As demonstrated in our study, bacterial resistance has developed against numerous antimicrobial agents. However, attributing this increase solely to the COVID-19 pandemic may be misleading. The WHO identified AMR in 2015 as a global threat that requires urgent action and included it in its Global Action Plan. AMR has even been referred to as a “slow pandemic” due to its profound threat to global health systems and its substantial economic burden [13]. Although AMR was already on the rise before the COVID-19 pandemic, the crisis may have accelerated this trend through excessive and inappropriate antibiotic prescribing, inadequate adherence to infection control protocols, patient care administered by inexperienced healthcare workers, and overwhelmed healthcare systems facing a surge in patient numbers [10, 13, 14]. These factors suggest that the pandemic served as a catalyst that amplified the existing crisis of AMR.

*Acinetobacter baumannii* is mainly isolated from the respiratory tracts of mechanically ventilated patients and has become a major hospital-acquired pathogen. Its remarkable ability to develop resistance, even to last-resort antibiotics, is supported by genetic plasticity, various adaptive mechanisms, and biofilm formation, which allow it to survive for months on inanimate surfaces. Outer membrane protein A (OmpA) further boosts colonization by mediating epithelial adhesion. Due to these characteristics, the WHO has classified *Acinetobacter baumannii* as a critical priority pathogen. In recent years, carbapenem resistance has become especially concerning, with prevalence rates reported at 76.2% in Asia, 69.4% in the Americas, and similarly high levels in the Mediterranean and Southern Europe [15]. In the present study, although the rise in carbapenem resistance among *Acinetobacter baumannii* isolates in the post-pandemic period did not reach statistical significance, an upward trend was observed. Furthermore, the resistance rate to carbapenems was also considerable, staying above 90%. Likewise, *Klebsiella pneumoniae* strains resistant to carbapenems have been reported to show an increasing trend since 2002, and this rise has accelerated during the pandemic, reaching resistance rates as high as 53% [2]. A review by Abbas et al. reported that *Klebsiella pneumoniae* has higher infection rates than other Gram-negative opportunistic pathogens. Its pathogenicity is supported by its ability to survive in nutrient-limited environments, intrinsic and acquired resistance, and the production of siderophores that promote iron acquisition and virulence. Aerobactin and yersiniabactin facilitate respiratory tract invasion and establishment of high bacterial loads, while enterobactin enhances lung colonization and dissemination. Adherence is further mediated by fimbriae, with type 3 fimbriae promoting biofilm formation and persistence. The review also noted that 50% of isolates were Extended-spectrum β-lactamases (ESBL)-producing and carbapenem-resistant [16]. However, our study revealed a more severe situation, with carbapenem resistance rates exceeding 70%.

Several post-pandemic studies have reported an increase in colistin and tigecycline resistance among *Acinetobacter baumannii* strains [2]. Colistin is considered the last line of defense against *Acinetobacter baumannii* infections; however, the increasing resistance rates pose a major global health concern [15]. These findings are consistent with the data obtained in our study. A similar trend was also observed for *Klebsiella pneumoniae*, where colistin resistance was reported to have increased during the COVID-19 pandemic, reaching up to 21.1% in some studies [17]. However, our findings indicate a far more concerning situation, with colistin resistance reaching a rate of 62.7%. The persistently high rates of carbapenem resistance may have led to an increased reliance on colistin and tigecycline in clinical practice. The observed rise in resistance to these agents is likely associated with this increased use.

During the COVID-19 pandemic, increased resistance to broad-spectrum antibiotics such as piperacillin-tazobactam, ceftazidime, and cefepime has been reported. Several studies have indicated that Gram-negative pathogens—particularly *Acinetobacter baumannii*, *Klebsiella pneumoniae*, and *Pseudomonas aeruginosa*—developed resistance to these agents during this period [8, 18]. In our study, although the increase in resistance against *Pseudomonas aeruginosa* was relatively modest compared to other Gram-negative bacteria, this pathogen remains one of the most important causative agents of healthcare-associated respiratory infections. Chronic colonization, especially in inflammatory airway diseases such as COPD, cystic fibrosis, and bronchiectasis, promotes the emergence of resistant strains. Additionally, the ability of *Pseudomonas aeruginosa* to form complex biofilms and the growth of microcolonies on the airway epithelium by biofilm aggregates are crucial in the development of resistance. According to the literature, resistance to carbapenems has been reported at a rate of 49.4% [19]. Our findings align with these reports, and a notable increase in carbapenem resistance was observed after the pandemic.

In our study, the isolation rate of *Escherichia coli* decreased from 11.4% before the pandemic to 7% afterward. Although mainly associated with urinary and gastrointestinal infections, *Escherichia coli* can also be found in respiratory samples, and its high resistance potential often requires treatment with third-generation cephalosporins, fluoroquinolones, or trimethoprim-sulfamethoxazole [20]. Although our analysis did not demonstrate a statistically significant increase in AMR rates against *Escherichia coli* during the post-pandemic period, upward trends were observed for several agents, including amikacin, trimethoprim-sulfamethoxazole, and tigecycline. Notably, published evidence indicates that resistance trends in *Escherichia coli* during the pandemic period exhibit substantial regional variability, underscoring the importance of local surveillance data [21]. Moreover, AMR rates of *Escherichia coli* vary based on patient age, sex, and sample source [22].

A recent study reported that resistance to third-generation cephalosporins in respiratory isolates of *Escherichia coli* exceeded 60%, aligning with our findings. However, the same survey found ciprofloxacin resistance at a rate of 50%, whereas in our group, this rate was significantly higher at 71% after the pandemic [22]. These increased resistance rates have been linked to the frequent use of cephalosporins and fluoroquinolones in empirical treatment regimens.

Among Gram-positive bacteria, *Staphylococcus aureus*, especially methicillin-resistant strains (*MRSA*), poses a significant global health threat. By infecting human epithelial cells, *MRSA* can suppress the host complement system, and its ability to form biofilms further boosts its survival. Compared to methicillin-susceptible strains, *MRSA* is linked to considerably higher morbidity and mortality, and it is estimated to cause around 30% of hospital-acquired infections [23, 24]. In this study, based on respiratory specimens, *MRSA* infection was found in 31.3% of cases before the pandemic and in 28% during the post-pandemic period, results that align with existing literature. Another species that can be isolated from respiratory specimens is *Enterococcus*. Although these bacteria are typically found in the human gastrointestinal tract, they are most commonly associated with infections of the lower urinary tract. In hospital-acquired infections, *Enterococcus faecalis* is identified as the primary species, accounting for approximately 80–90% of cases, while *Enterococcus faecium* is less common, responsible for roughly 10–15% of infections. A recent study reported vancomycin resistance rates of 2.7% for *Enterococcus faecalis* and 41.2% for *Enterococcus faecium* [25]. Our results differ from these findings, possibly because our study only included respiratory isolates and involved a relatively small number of samples.

According to 2015 data, Türkiye was identified as the Organisation for Economic Co-operation and Development (OECD) member country with the highest AMR rate, at 38.8%. Although the Ministry of Health has taken some measures to address this issue, the anticipated decrease in resistance levels has not been achieved. In a report from Turkey, İşler et al. highlighted three urgent priorities to reduce AMR. The first is the development of a national action plan for infection prevention and control against multidrug-resistant organisms; the second is the implementation of stricter measures regarding antibiotic use in hospitals; and the third is identifying resistance patterns in community-acquired infections, along with health policy strategies and public awareness campaigns. Additionally, the role of agriculture and livestock in contributing to AMR in Türkiye remains unclear, underscoring the need for further research and the adoption of a “One Health” approach to combat resistance [26].

Preventive measures can also be implemented at the hospital level. In our long-standing tertiary care hospital, improvements include upgrading physical infrastructure, addressing staffing shortages, providing training on hand hygiene and other infection prevention strategies, and increasing the active involvement of the infection control team as observers and supervisors throughout the hospital, including outpatient clinics. These control measures, which have been increasingly neglected during the COVID-19 pandemic, should be urgently reintroduced. Additionally, collaboration not only with the Ministry of Health but also with other organizations and international partners would help establish a more effective surveillance system.

Following the COVID-19 pandemic, a marked increase in AMR rates has been observed worldwide. This study makes a significant contribution to the literature, as it is one of the few quantitative analyses conducted in Turkey on this subject.

### Limitations

This study has several limitations. First, the specific genetic determinants responsible for AMR in the isolated pathogens were not identified, which limits the ability to explore underlying resistance mechanisms. Second, the study was conducted in a single tertiary care center in one region, which may limit the generalizability of the findings to other healthcare settings or geographic locations. Additionally, it was not possible to determine which patients received steroid therapy, the dosage administered, or whether steroids were given in the emergency department, which represents another limitation of the study. And also, the lack of data on other viral infections is another limitation of the study.

## Conclusion

The findings of this study indicate that AMR in intensive care units is not only a problem during the pandemic but also represents a worsening crisis that has accelerated since its onset. The increasing resistance rates are concerning for infection management and indicate a shrinking range of treatment options for hospital-acquired infections. The loss of susceptibility to each antibiotic poses a threat to both current treatments and future options. This study is particularly important because it provides quantitative insights into how the global crisis affects Turkey. Additionally, identifying risk factors that lead to higher AMR requires well-designed, prospective, multicenter studies.

## Data Availability

The datasets used and/or analyzed during the current study are available from the corresponding author on reasonable request.

## Abbreviations

AMR: Antimicrobial Resistance
WHO: World Health Organization
ICU: Intensive Care Unit
COPD: Chronic Obstructive Pulmonary Disease
BAL: Bronchoalveolar Lavage
DTA: Deep Tracheal Aspirate
DM: Diabetes Mellitus
HT: Hypertension
CAD: Coronary Artery Disease
CHF: Chronic Heart Failure
CLSI: Clinical and Laboratory Standards Institute
EUCAST: European Committee on Antimicrobial Susceptibility Testing
MIC: Minimum Inhibitory Concentration
SPSS: Statistical Package for the Social Sciences
OmpA: Outer Membrane Protein A
ESBL: Extended-Spectrum β Lactamases
MRSA: Methicillin-Resistant Staphylococcus Aureus
OECD: Organisation for Economic Co-operation and Development
